# Redox signaling-governed drug-tolerant persister cancer cell: a key spark of treatment failure

**DOI:** 10.1038/s41392-022-00940-0

**Published:** 2022-03-17

**Authors:** Liwu Fu, Zhe-Sheng Chen

**Affiliations:** 1 State Key Laboratory of Oncology in South China, Collaborative Innovation Center for Cancer Medicine, Guangdong Esophageal Cancer Institute, Cancer Center, Sun Yat-Sen University, Guangzhou, 510060 China; 2 Department of Pharmaceutical Sciences, College of Pharmacy and Health Sciences, St. John’s University, Queens, NY 11439 USA; 3 Institute for Biotechnology, St. John’s University, Queens, NY 11439 USA

**Keywords:** Cancer therapy, Cancer models

In a recent study published in *EMBO Molecular medicine*, Zhang et al. provided a novel insight regarding better knowledge of the drug resistant contribution of drug-tolerant persister (DTP) state in tumor evolution, especially in terms of mechanistic insights into redox regulation involved, unambiguously establishing a novel therapeutic strategy of treating multi-drug-resistant (MDR) cancer cells and preventing tumor recurrence by disequilibrating Niemann–Pick C1-like 1 (NPC1L1)-mediated redox homeostasis.^1^

**Fig. 1:**
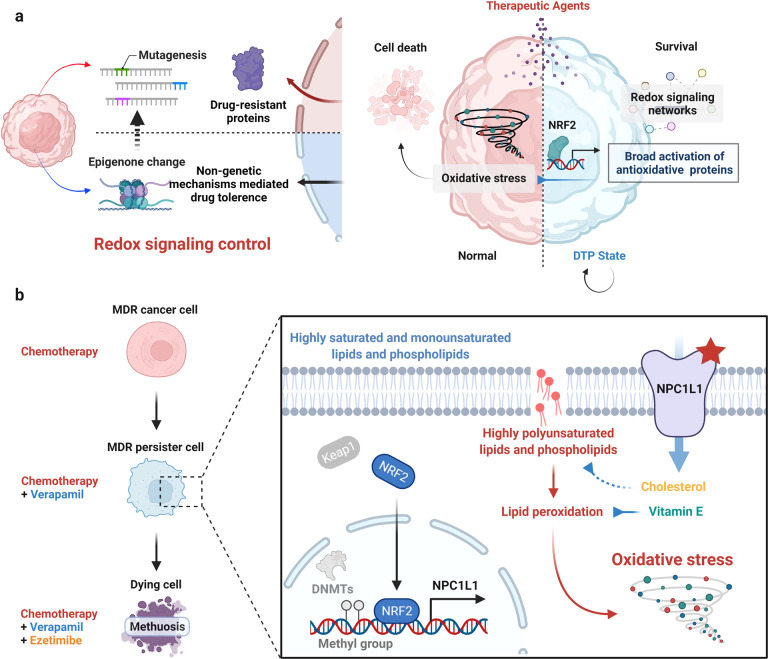
A drug-tolerant persister (DTP) state enables multidrug resistant (MDR) cancer cell survival after the canonical MDR reversal treatment. **a** Variable levels of drug resistance in cancer cells are attributed to genetic heterogeneity, which induces the high expression of drug-resistant proteins, thus leading to MDR clone formation. However, nongenetic variations (epigenetics) in cancer cells can occur upon cancer therapy, representing the source of genetically resistant clones and resulting in several unusual changes including a highly flexible metabolic pathway, the slowdown of proliferation, stemness maintenance *etc*. These phenotypes hold extensive overlaps with embryos under stressful environments and are closely linked to the modulation of redox signaling. Indeed, cancer cells entering a DTP state can reset the redox signaling networks, therefore possessing an antioxidative defense mechanism against oxidative stress. **b** Zhang et al. present the detailed mechanisms for illuminating the role of redox balance in persisters. The figure was created by BioRender

The phrase “a single spark can start a prairie fire” aptly describes the role of DTP cancer cells in anticancer-treatment failure. DTP cancer cells implement globally transcriptional reprogramming by activating a nongenetic mechanism, therefore escaping therapeutic stress and eventually triggering a set of serious outcomes after drug withdrawal, including drug resistance and tumor relapse.^2^ Remarkably, DTP cancer cells exhibit significant similarities to embryos under stress, including the distinct metabolic pattern, stemness maintenance, and transient cell cycle arrest.^3^ These features have inextricable links to the orchestration of redox signaling, which enables DTP cancer cells to survive constant stress over the course of treatment by developing adaptive mechanisms to avoid endogenous or exogenous oxidative stress (Fig. 1a). However, the detailed mechanisms that drive powerful antioxidant systems of DTP cancer cells are not clear, and existing available agents based on redox regulation, such as glutathione peroxidase 4 (GPX4) inhibitors, remain challenging to translate into therapeutic uses. To this end, Zhang and colleagues,^1^ focusing on redox status of the DTP cancers from MDR cancer cells, identified a novel vulnerability—NPC1L1, a specific target of an antihyperlipidemic drug that has been approved by the United States Food and Drug Administration, ezetimibe. Their study illuminates the role of redox signaling in regulating the survival of DTP cancers and confirms the potential of targeting NPC1L1 by ezetimibe for treating MDR disease, thereby delaying cancer recurrence (Fig. 1b).

First, Zhang and colleagues evaluated whether human MDR cancer cells possessed the cell-state plasticity for entering DTP state during canonical MDR-reversal treatment using a combination of chemotherapeutic agents and MDR1 inhibitor verapamil. Zhang and colleagues found that a small cluster of MDR cancer cells developed DTP phenotypes after only three days of treatment (exome sequencing between pre- and post-treated MDR cancer cells was performed for excluding genetic resistance). In contrast, DTP phenotypes in parental nonresistant cancer cells were gradually observed after a 9-day treatment. These results indicated that either MDR or nonresistant persisters will emerge sooner or later during treatment and are consistent with the recent view proposed by Rehman et al.^3^ that “all tumor cells have equal capacity to enter and exit the DTP state to survive therapy”. In addition, their findings also imply that the evolution speed is likely dependent on the original integrity of antioxidant systems, as MDR population can mobilize a set of antioxidant mechanisms more rapidly than nonresistant cancer cells, which not only trigger cell proliferation but also cope with oxidative stress induced by anticancer agents.

To characterize the mechanisms underpinning MDR persister survival, the authors used RNA sequencing to compare the transcriptional profile of MDR cancer cells and MDR persister cells. They identified NPC1L1, a well-known sterol transporter, as a major modulator increased in MDR persister based on the consistency and prominence of transcriptome results. By inducing excessive oxidative stress, NPC1L1 inhibition increased treatment cytotoxicity and sharply decreased the number of persisters, whereas recovering the key substrates of NPC1L1, cholesterol, and vitamin E, markedly compromised lipotoxicity-mediated oxidative stress. Indeed, the notion that inhibiting NPC1L1 could eliminate MDR cancer cells is intriguingly interlinked with previous studies. For instance, Oren et al.^4^ recently established the role of activated fatty acid metabolism in supporting persister growth, and NPC1L1-mediated cholesterol absorption is likely involved in this process. Moreover, persister cells characterized by the accumulation of highly polyunsaturated lipids and phospholipids are widely recognized as exquisitely sensitive to ferroptosis induced by excessive accumulation of lipid peroxide.^5^ Thus, NPC1L1-mediated cholesterol and vitamin-E absorption may be a potential approach for persisters to alleviate ferroptosis. However, Zhang and colleagues did not focus their attention to ferroptosis, as they observed an unusual cytoplasmic vacuolization that was later identified as methuosis (excessive macropinocytosis) followed by NPC1L1 inhibition using ezetimibe. This special biological process, similar to autophagy, is advantageous for survival by indiscriminately enhancing nutrient absorption in the early period. Yet, the counterintuitive macropinocytosis induced by ezetimibe treatment eventually progressed to methuosis, thus promoting the absorption of anticancer drugs and inducing a catastrophic and rapid fluid uptake.

Recognizing that NPC1L1 plays a unique role in maintaining redox balance of persisters, Zhang and colleagues next investigated how therapeutic stress induces the expression of NPC1L1. The authors found that nuclear factor erythroid-2-related factor 2 (NRF2) as a main transcription factor in response to oxidative stress was significantly induced and bound to the promoter of NPC1L1, therefore transcriptionally contributing to NPC1L1 expression. Furthermore, the reversible DTP state is mediated by epigenetics and the CpG island of the NPC1L1 gene is usually epigenetically silenced in many tissues. Thus, the authors also noted that DNA methyltransferase-induced NPC1L1 silence was reduced in MDR persister cancer cells.

In line with the context in the field, the work by Zhang and colleagues highlights the significance of nongenetic redox resetting in persister-cell survival and confirms the remarkable anticancer effect of the combination of chemotherapeutic agents, verapamil, and NPC1L1 inhibitor ezetimibe. Notably, the clinical relevance of their finding is immediate, given that the NPC1L1 inhibitor ezetimibe is prescribed widely for hypercholesterolemia without major side effects. These encouraging findings suggest that modulating the redox balance in persisters could eliminate the cluster of malignant cells, thereby avoiding further drug resistance and cancer recurrence.
